# Mapping Late Holocene Vegetation Change Using Isopollen Analysis: Evidence from the Southeastern Marmara Region, Türkiye

**DOI:** 10.3390/plants15121881

**Published:** 2026-06-17

**Authors:** Çağlar Altıncı, Gülan Güngör, Hülya Caner

**Affiliations:** 1 Institute of Earth and Marine Sciences, Gebze Technical University, Gebze 41470, Türkiye; caglaraltinci@gmail.com; 2 Institute of Social Sciences, Istanbul University, Istanbul 34452, Türkiye; gulangungor3636@gmail.com; 3 Department of Marine Geology and Geophysics, Institute of Marine Sciences and Management, Istanbul University, Istanbul 34452, Türkiye

**Keywords:** fossil pollen, isopollen mapping, human–environment interactions, vegetation dynamics

## Abstract

Determining the relative impacts of climate variability and human activities on vegetation dynamics remains a central theme in paleoecological research. In climate transition zones like the southeastern Marmara region, isopollen maps are important because they allow for the evaluation of spatially diverse pollen records within an integrated regional framework. The aim of this study is to present a spatially holistic reconstruction of Late Holocene vegetation change in the southern Marmara region using isopollen maps based on fossil pollen records obtained from Manyas, Iznik and Sapanca lakes. Isopollen maps were created for five time periods, approximately 2600, 2000, 1250, 800 and 400 yr BP, representing major climatic and historical phases of the Late Holocene, and the spatial distribution patterns of the major tree and herbaceous taxa were reconstructed. The results demonstrate the presence of a continuous west–east variability in the region’s vegetation structure, reflecting the transition between Mediterranean and Black Sea climate regimes. However, the temporal variation patterns show that vegetation responses cannot always be directly explained by climatic phases. In particular, *Artemisia* highlights the persistence and local expansion of open-area vegetation, reaching approximately 24% of the study area to the present day. Given the region’s long history of settlement, these findings indicate that vegetation dynamics during the Late Holocene were shaped by the combined effects of climatic changes, local environmental conditions and human activities. Therefore, the study emphasizes the importance of spatially integrated approaches in paleoecological reconstructions.

## 1. Introduction

Fossil pollen analyses, as one of the most important sub-disciplines of Quaternary paleoclimatology, provide continental-scale data that complement information obtained from marine sediments and ice cores [1,2,3]. Fossil pollen analyses are widely used worldwide, particularly for reconstructing vegetation responses to climate change, revealing phytogeographic distribution patterns of specific taxa, and estimating past climate parameters. Through these analyses, not only natural environmental changes but also the scale and time intervals at which human impact transformed vegetation dynamics can be determined. In identifying human impact in pollen diagrams, numerous paleoenvironmental indicators are considered together, such as changes in the overall composition of vegetation, the increase in indicator species, the decline of forest taxa, and the emergence of pollens from cultivated plants [4,5]. Reconstructing vegetation is one of the fundamental aims of Quaternary paleoecology and palynology. Vegetation on Earth directly affects many aspects of the environment, including climate, food resources, and the quality and accessibility of water. Understanding past processes contributes to a more accurate determination of which processes should be included in climate, dynamic vegetation, and watershed models used to predict future changes [6].

Fossil pollen analyses performed on sediment cores from different accumulation basins in Anatolia are increasing; in parallel, the need for reassessment of paleovegetation and paleoclimate conditions in Anatolia as a whole or on a regional scale is becoming more apparent. The combined analysis of fossil pollen data obtained from accumulation environments in different locations allows for the evaluation of paleovegetation and paleoclimate changes on a temporal and spatial scale. However, studies in this scope are still quite limited for Anatolia, and the first study on vegetation reconstruction covering the whole of Anatolia was carried out by van Zeist and Bottema [7]. In this study, paleovegetation maps were created for selected periods from 18,000 to 16,000 years, 12,000 to 11,000 years, 8000 years, and 4000 years ago using pollen diagrams obtained from different regions of Anatolia.

McMillan [8], on the other hand, developed a vegetation modelling framework to explain the paleovegetation characteristics of southwestern Türkiye. In this context, high-resolution bioclimatic modelling, Bayesian chronology modelling based on pollen zones and radiocarbon dates, and simulation approaches in which vegetation outputs are converted into pollen signals have been used together. Şenkul and Köse [9] also re-evaluated fossil pollen records from the last 2000 years in the Cappadocia region through a holistic approach using geographic information system (GIS)-based isopollen maps, revealing the temporal and spatial changes in selected plant taxa under paleovegetation conditions and the possible factors influencing these changes. While these studies make significant contributions to the regional-scale evaluation of fossil pollen data in Anatolia, spatial and holistic interpretations regarding the southeastern part of the Marmara Sea still need to be developed.

The main aim of this study is to evaluate the climatic changes and anthropogenic impacts that occurred during the Late Holocene in the southeastern part of the Marmara Sea from a spatial perspective, to reveal the reflections of these processes on vegetation, and to interpret these environmental transformations by visualizing them through isopollen maps. Isopollen maps are thematic isorhythmic maps that spatially represent pollen distribution, created by combining pollen data based on equal density and distribution curves. These maps, which can be produced using current or fossil pollen data, show that the center with the highest isopollen indicates the area where the relevant pollen is represented with the highest frequency. Beyond visualizing pollen distribution, isopollen maps also contribute to understanding spatial and temporal changes in vegetation, as well as the impact of human activity on vegetation [10].

## 2. Materials and Methods

### 2.1. Study Area

**Figure 1:**
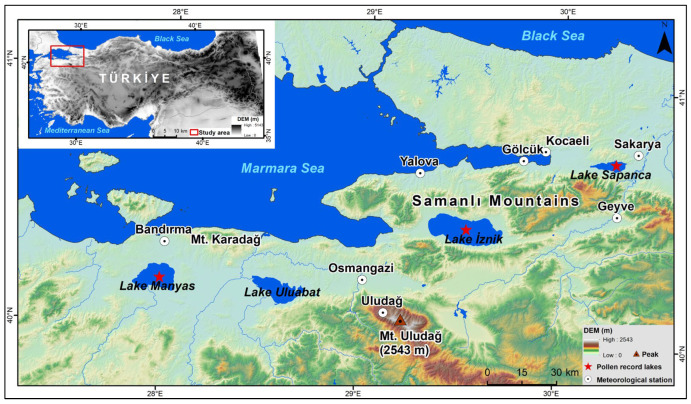
Location of pollen record lakes and meteorological stations used in the study in the southeastern Marmara region.

This study, conducted to spatially visualize and analyze pollen density obtained from fossil pollen analyses, utilized data from fossil pollen analysis studies carried out in Manyas, Iznik, and Sapanca lakes. The selected lakes are in the southeastern part of the Marmara Sea, bordered by the Kocaeli Plateau to the north, the western part of the Kapıorman Mountains to the east, the Uludağ massif to the south, and the Gönen Basin to the west (Figure 1). The highest point of the Samanlı Mountains, a significant elevation in the study area, is Kartepe (1602 m), located in the northeast.

To determine the spatial distribution of temperature and precipitation conditions in the study area, WorldClim temperature data from Hijmans et al. [11] were used. The average annual temperature generally decreases from the coast towards the interior. Humidity, continentality, and altitude are factors influencing this. In Uludağ, the eastern part of the Samanlı Mountains, the northern part of the Sündiken Mountains, and the Kapıorman Mountains, the increase in altitude and continentality lead to a decrease in temperature. According to the total annual precipitation, the areas with the highest precipitation are the northern part of the İzmit Gulf and Lake Sapanca, as well as Uludağ and the Samanlı Mountains. The study area is generally located within the transitional climate zone of the Marmara region, which lies between the Mediterranean and Black Sea climate zones. While the influence of the Mediterranean climate is more pronounced around Manyas and Iznik lakes, the influence of the mild and humid Black Sea climate is more pronounced around Sapanca Lake and its surroundings due to increased autumn rainfall.

According to the data obtained from the meteorological stations located in the study area, the Köppen–Geiger climate classification was applied using eight stations (Figure 1). One of the most important features of Köppen–Geiger climate classification is that climate types are compatible with the distribution of plant communities. In the study area, a mid-latitude climate with cold winters is observed in areas where elevation increases, such as Uludağ and the Samanlı Mountains. While a warm temperate climate with precipitation in all seasons is observed in the northeast of Lake Sapanca, a Mediterranean climate, characterized by mild winters and hot, dry summers, is observed across most of the study area. Among the lakes where fossil pollen analyses were conducted, Lakes Manyas and Iznik are located within the Mediterranean climate zone, whereas Lake Sapanca is situated between the Mediterranean climate zone and the Black Sea climate zone, which is characterized by a warm temperate climate with precipitation in all seasons.

The study area is in a region exhibiting transitional characteristics between the European–Siberian and Mediterranean floristic regions. The humid forests found in the European–Siberian floristic region are referred to as the euxine province in our country and are characterized by climax beech (*Fagus orientalis*) forests [12]. In terms of plant communities, the study area can be divided into humid and semi-humid forests, dry forests, maquis and pseudo maquis areas, and alpine vegetation areas. The distribution area of humid forests is mainly the north-facing slopes of mountainous areas. In the mountainous areas of the study area, the northern parts of Uludağ and the Samanlı mountains, and the Kapıdağ and Karadağ massifs are particularly favorable areas for the development of humid forests in terms of rainfall amount, distribution of rainfall over months, and temperature conditions. In areas without destruction, humid forests start immediately from the coastline, while in areas with destruction, they start after shrub groups and rise upwards [13,14]. In the Samanlı Mountains, humid forests extend from the upper part of the pseudo maquis to the upper levels of the mountain. The main dominant elements of humid forests are the species *Fagus orientalis*, *Quercus petraea*, *Castanea sativa* and *Tilia tomentosa*. In the study area, humid forests, starting behind the pseudo maquis formation, are represented by *Castanea sativa* and *Quercus petraea* at lower altitudes, and by *Fagus orientalis* at higher altitudes, up to 900–950 m in the western part of the Samanlı massif, 1200 m in the central part, 1600 m in the eastern part, and 1700–1800 m in Uludağ, while *Abies bornmülleriana* is represented at higher levels in Uludağ. In Karadağ, after the pseudo maquis formations located on the coast, *Quercus petraea* forests are found at lower altitudes, *Castanea sativa* forests at higher altitudes and *Fagus orientalis* forests at even higher altitudes [13].

Dry forests are the dominant plant community in the study area, on the southern slopes of mountainous regions, inland plateaus and hills. Composed of drought-resistant trees and shrubs, these forests differ significantly from humid forests in their species poverty, lack of undergrowth, and sparse appearance. Dry forests on the southern slopes of mountainous regions are generally represented by drought-resistant oak species (the dominant element being *Quercus infectoria*) at lower altitudes, *Pinus nigra* in the central regions, and *Pinus sylvestris* in some higher altitudes. In the hilly and plateau areas of the interior, *Pinus nigra* is the dominant species, while *Pinus brutia* and *Quercus infectoria* are dominant on the low hills and plateaus inland [13].

In the study area, maquis and pseudo maquis are primarily distributed in coastal regions and valleys where the sea influence has penetrated, after the forest has been cleared. While pseudo maquis is more commonly found in coastal regions where humid forests are prevalent, maquis is distributed in coastal areas where dry forest areas on the southern slopes have been destroyed. The most common maquis species in the study area are *Phillyrea latifolia*, *Pistacia terebinthus*, *Quercus coccifera*, *Juniperus oxycedrus*, *Erica arborea*, *Arbutus unedo* and *Cistus creticus*. The pseudo maquis species found in the study area include maquis elements such as *Arbutus unedo*, *Erica arborea*, *Phillyrea latifolia*, *Pistacia terebinthus*, *Cercis siliquastrum* and *Spartiun junceum*, as well as moisture-loving species such as *Fraxinus ornus*, *Corylus avellana*, *Cornus mas*, *C. sanguinea*, *Prunus spinosa* and *Sorbus torminalis*. Deciduous, hygroscopic species dominate the pseudo maquis formation, while evergreen, drought-tolerant plant species are dominant in the maquis formation [13].

Alpine vegetation is found above the forest line, and in the study area, plant species belonging to this formation are only found in the Uludağ massif. The alpine zone, which begins after 2100 m, where the fir forests end in Uludağ, is widespread up to 2200–2300 m with *Juniperus nana*, and from this altitude to the peaks, *Festuca punctoris*, *Astragalus* subsp., *Asperula nitida* and *Acantholimon* subsp. are common [13].

Some settlements in the study area date back to the Neolithic period. Humans began to develop a lifestyle dependent on changes in natural environmental conditions during the Neolithic period [15]. Among the earliest examples of settlements in the region are Menteşe Höyük around Lake Iznik, one of the oldest settlement areas in the study area, and Barcın Höyük, Ilıpınar, and Aktopraklık settlements in Bursa. During the Chalcolithic Period, which followed the Neolithic period, village settlements grew and became more fortified, and in the subsequent period, the foundations of the first cities were laid. Among the ancient cities in the study area, the most densely populated and important centers are Nicaea (Iznik) and Nicomedia. In later periods, the region was successively under the rule of the Roman, Byzantine, and Ottoman empires.

### 2.2. Construction of Isopollen Maps

Fossil pollen data from Manyas, Iznik, and Sapanca lakes, included in the Neotoma Paleoecology Database, were used to determine the Late Holocene paleovegetation conditions in the southeastern part of the Marmara Sea (Table 1) [16,17,18]. The lakes included in the study were selected based on the presence of comparable plant taxa in fossil pollen studies and the use of radiocarbon dating techniques in those studies.

To assess climate change and anthropogenic impacts, specific indicator plant species were selected. Data obtained from the Neotoma Paleoecology Database were first grouped as herbaceous (non-arboreal, NAP) and woody (arboreal, AP) pollens, and then the AP group was converted into percentage values. The same process was applied to the NAP group. In the final stage, the percentage distribution of AP and NAP pollens within the total pollens was calculated. In addition, specific indicator plant species were selected to assess climate change and anthropogenic impacts. The indicator species used in the study were identified as *Pinus*, *Quercus*, *Fagus*, *Alnus*, *Artemisia* and Poaceae [19,20]. Therefore, isopollen maps are presented in pairwise combinations according to the ecological and paleoenvironmental significance of the selected taxa. *Pinus* and *Quercus* are grouped as the main woodland/forest taxa, *Fagus* and *Alnus* as indicators of relatively humid environmental conditions, and *Artemisia* and Poaceae as indicators related to open areas and degradation. These species are plant groups known to reflect different climatic conditions and human impacts and are widely used in paleoecological studies. For example, *Pinus* and *Quercus* indicate the prevalence of forest cover, while *Artemisia* and Poaceae species are associated with more open areas, agricultural fields, and steppe-like habitats. *Fagus* and *Alnus* were considered indicators of humid climatic conditions. Furthermore, the increase in species such as Poaceae (especially cereal pollens) and *Artemisia* may point to land use changes and agricultural activities due to human activities. The time interval selected for reconstruction in this study was determined as the last 3000 years, representing the Late Holocene, based on the common radiocarbon-dated interval of the lakes with available fossil pollen data. To facilitate easier connection with climate change, the chronology in this study is shown with isopollen maps for five different dates: 400 yr BP (Little Ice Age), 800 yr BP (Medieval Warm Period), 1250 yr BP (Migration Period), 2000 yr BP (Roman Warm Period) and 2600 yr BP (Iron Age Cold Period), according to the cold and warm periods within the Late Holocene [21]. Isopollen maps were generated to reconstruct the spatial distribution of selected taxa for each time. Pollen percentage values were extracted and standardized prior to spatial analysis. Spatial interpolation was performed using the Inverse Distance Weighting (IDW) method to generate continuous surfaces from point-based pollen data. This method assumes that closer observations have a greater influence on interpolated values than more distant observations and is therefore suitable for datasets with limited spatial coverage. However, due to the limited number of sampling areas, the resulting interpolated surfaces should be interpreted as generalized spatial trends rather than precise spatial representations. The reason for the limited number of sampling areas stems from the limited availability of fossil pollen datasets for the study area in the Neotoma Paleoecology Database, and only records with comparable taxa, radiocarbon dating verification, and a common Late Holocene chronological range were used in the analysis. All spatial analyses and mapping processes were performed using GIS software ArcGIS 10.4 (ESRI, Redlands, CA, USA). Interpolated raster surfaces were generated at consistent spatial resolution, cropped according to study area boundaries, and categorized into graded ranges to visualize relative changes in pollen abundance. Final isopollen maps were generated for each time and taxon. This made it possible to compare spatial patterns in vegetation dynamics during the Late Holocene and their relationship to climate variability and human-induced impacts.

## 3. Results

### 3.1. Pinus

**Figure 2:**
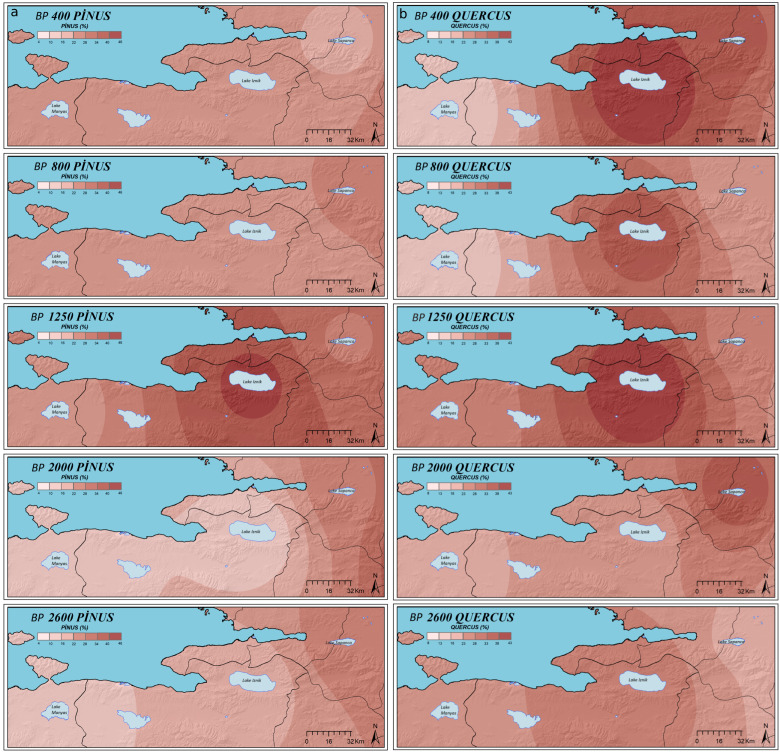
(**a**,**b**) Spatial distribution of *Pinus* and *Quercus* pollen percentages in the southeastern Marmara region during the Late Holocene.

*Pinus* is represented in the study area by *Pinus nigra* and *Pinus brutia* in large areas, and by *Pinus sylvestris* and *Pinus pinea* in smaller areas [22]. Although the amount of pollen production varies among each genus and species, *Pinus*, an anemogamous plant species, produces a large amount of pollen, and the percentages of *Pinus* pollen among woody species are high in both fossil and current pollen data [23]. This situation is observed in all fossil pollen data of the selected lakes. In the fossil pollen data of the three selected lakes, the percentage of *Pinus* pollen varies between 4% and 45% (Figure 2a). In the study area, except for the 400 yr BP, *Pinus* density generally decreases from east to west in all time intervals. The lowest *Pinus* pollen percentage is observed in a warm period before 2000 yr BP, varying between 4% and 10% around Lake Manyas and Lake Iznik. However, the percentage of *Pinus* pollen increases towards the east and is distributed between 22–28% in Lake Sapanca. The highest *Pinus* pollen percentage is reached at 1250 yr BP, a cold period, at 45% (Figure 2a). During this period, *Pinus* is found at 30% and higher in the east of the study area, with its densest distribution around Lake Iznik. Subsequently, when the isopollen maps showing the years 800 and 400 yr BP are examined, a decrease in *Pinus* pollen density is observed. In 400 yr BP, the *Pinus* rate is higher in the west, around Lake Manyas and Lake Iznik, unlike other periods.

### 3.2. Quercus

*Quercus* is a genus of the Fagaceae family, distributed in temperate and subtropical regions with numerous species. It is a woody plant found as deciduous or evergreen trees and shrubs [24]. While deciduous oak species tolerate low winter temperatures, they require high temperatures during their growth period [25]. In the study area, it is represented by species such as *Quercus coccifera*, *Quercus cerris*, *Quercus infectoria*, *Quercus petraea* and *Quercus frainetto*. In the study area, *Quercus petraea* forms stands with *Fagus*, *Castanea* and *Tilia* trees, especially in humid forests; and with *Pinus nigra* trees in semi-humid and semi-arid forests.

When the isopollen maps of *Quercus* are examined, it is seen that *Quercus* varies between 8% and 43% spatially and temporally. Isopollen maps show that the proportion of *Quercus* pollen is particularly higher around Lake Iznik than Lake Sapanca, compared to Lake Manyas. Within the isopollen maps, regional *Quercus* pollen concentrations are generally lower during warm periods (2000–800 yr BP), ranging between approximately 8% and 38%, compared to other periods, and relatively higher during cold periods (1250–400 yr BP), ranging between approximately 8% and 43%. The lowest *Quercus* pollen percentage is observed in the study area in 2600 yr BP at 18–28%, and even then, it is more concentrated around Lake Iznik than in other areas. In 400 yr BP, Lake Iznik has the highest concentration, reaching up to 43%, followed by Lake Sapanca and finally Lake Manyas (Figure 2b).

### 3.3. Alnus

**Figure 3:**
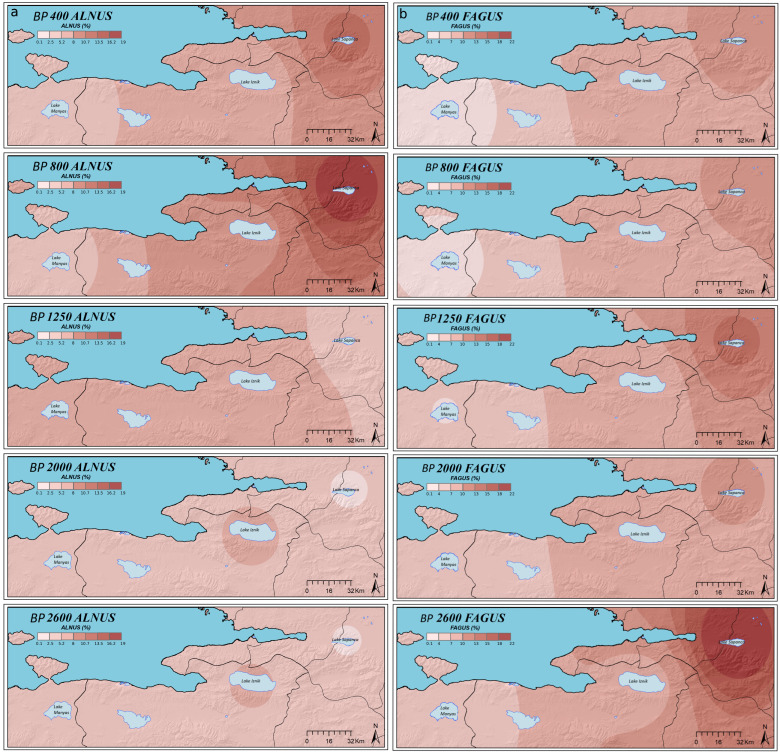
(**a**,**b**) Spatial distribution of *Alnus* and *Fagus* pollen percentages in the southeastern Marmara region during the Late Holocene.

*Alnus*, being a hygroscopic plant, is currently located within the humid forest area facing north in the study area [13,14]. When isopollen maps of *Alnus* are examined, it is represented in the study area with a pollen ratio varying between 0.1% and 19%. Examination of isopollen maps shows that *Alnus* pollen ratios are lower in the study area at 2600 and 2000 yr BP. The percentage of *Alnus* towards the present day then begins to increase, reaching its highest rate in the study area at 800 yr BP at 19% in Lake Sapanca (Figure 3a). Although this increase decreases to 400 yr BP, it remains relatively high around Lake Sapanca compared to other areas.

### 3.4. Fagus

In the current vegetation of the study area, humid forests are located on north-facing slopes or in troughs that allow moisture penetration due to the influx of humid air masses from the north. Examination of isopollen maps shows that *Fagus* pollen concentrations vary between 0.1% and 22%. Generally, *Fagus* concentrations increase from west to east. The area with the lowest *Fagus* concentration throughout all time periods is Lake Manyas and its surroundings, while the area with the highest *Fagus* concentration is Lake Sapanca and its surroundings. Within the isopollen maps, the highest *Fagus* distribution in the study area is observed at 2600 yr BP (Figure 3b). The lowest *Fagus* pollen density is seen in the isopollen map of 400 yr BP, the most recent period (Figure 3b). Overall, it is observed that *Fagus* pollen density in the study area has decreased over the years.

### 3.5. Artemisia

*Artemisia*, which naturally grows in the Iran–Turan phytogeographic region of our country, is an herbaceous species with steppe characteristics [26]. As a natural vegetation product of continental climate zones, *Artemisia* is also an important indicator of abandoned or fallow agricultural areas [19]. Furthermore, *Artemisia* is considered an indicator species during cold and dry periods [26,27].

**Figure 4:**
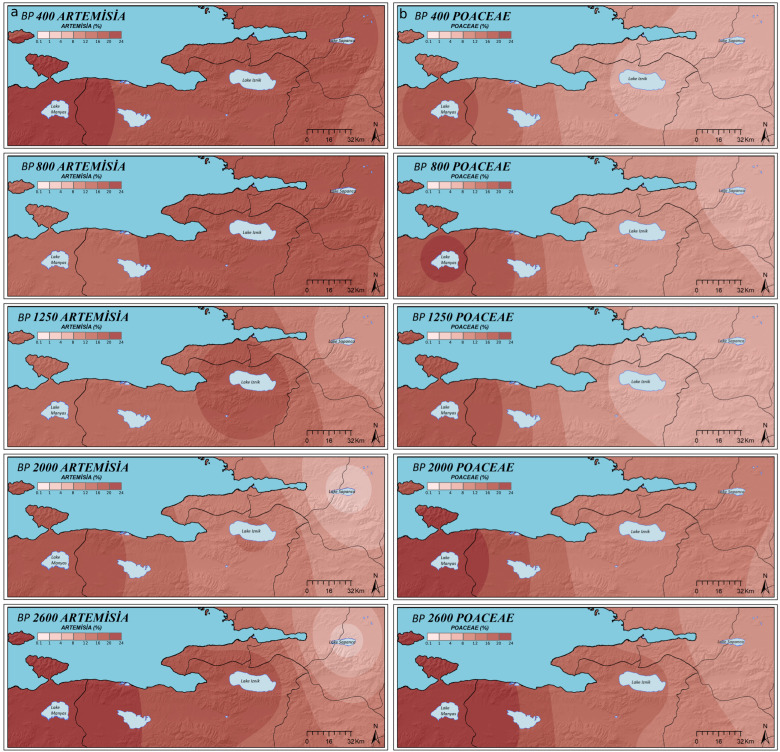
(**a**,**b**) Spatial distribution of *Artemisia* and Poaceae pollen percentages in the southeastern Marmara region during the Late Holocene.

Examination of isopollen maps shows that *Artemisia* pollen is represented in the study area in percentages ranging from 1% to 24%, varying spatially and temporally. Due to its status as a natural vegetation of the continental climate, *Artemisia* pollen is generally found in high concentrations around Lake Manyas due to climatic conditions, while Lake Sapanca and its surroundings, being located within the euxinian floristic region, have lower concentrations of this type of pollen. Within the isopollen maps, the period with the lowest *Artemisia* pollen concentration corresponds to approximately 2000 years ago, with values decreasing to 0.1%, especially around Lake Sapanca (Figure 4a). During cold climate periods such as 2600, 1250 and 400 yr BP, *Artemisia* pollen concentrations are higher than in other periods. Generally, as we approach the present day, the pollen density of *Artemisia* pollen begins to increase in and around Lake Sapanca.

### 3.6. Poaceae

Poaceae, along with *Artemisia* and Chenopodiaceae, are herbaceous taxa generally considered indicators of steppe vegetation [28]. Poaceae includes many economically important plants, such as *Zea*, *Triticum*, *Hordeum*, *Secale* and *Avena*. Examination of isopollen maps shows that Poaceae pollen ratios vary between 1% and 24% across time and space in the study area. Overall, a decrease in Poaceae pollen ratios from west to east is observed across all time periods in the study area. Examination of isopollen maps from 2600 yr BP to 2000 yr BP reveals the highest Poaceae distribution around Lake Manyas, where values reach up to approximately 24%. Although there is a decrease in Poaceae ratio around 1250 yr BP, the densest distribution is still observed around Lake Manyas. Although Poaceae pollen ratios appear to increase to 800 yr BP compared to the previous period, the isopollen map from 400 yr BP shows a decrease in Poaceae pollen ratio (Figure 4b). The spatial distribution of Poaceae pollen is mainly concentrated in the western part of the study area, around Lake Manyas and during periods of higher concentration, it extends eastward approximately 130 km towards the Lake Iznik basin.

## 4. Discussion

The integration of fossil pollen records from Manyas, Iznik and Sapanca lakes provides a comprehensive framework for assessing vegetation dynamics in the southeastern Marmara region during the Late Holocene. The results suggest that vegetation change cannot be attributed to a single climatic factor but rather reflects the combined effect of regional climate gradients, local environmental controls, and increasingly intensified anthropogenic pressure. This interpretation is consistent with broader paleoecological syntheses in the Mediterranean basin examining vegetation change during the Holocene [25,29]. Due to the location of the study area, a distinct west–east spatial divergence emerges, reflecting the transitional nature of the Marmara region between the Mediterranean and Black Sea climate systems. In the western part, represented by Lake Manyas, the dominance of steppe-related taxa such as *Artemisia* and Poaceae indicates more continental and relatively drier conditions. Although most of the tree taxa selected for the study are primarily associated with C3 photosynthesis, the distribution of Poaceae may also be partly related to differences in photosynthetic strategies, particularly in warmer, drier, and more open environmental conditions [30]. In contrast, the eastern part, including the Sapanca and Iznik lake basins, is characterized by higher proportions of mesophilic and hygrophilous taxa such as *Fagus* and *Alnus*, reflecting increased humidity and possible strong maritime influence. This spatial pattern reflects current vegetation distributions and shows that regional climatic gradients exerted continuous control over vegetation structure throughout the Late Holocene, consistent with large-scale pollen syntheses across Europe and Anatolia [29,31].

Examination of the isopollen maps of indicator plants in the study area reveals that *Fagus* shows a significant density, particularly around Lake Sapanca, in 2600 yr BP. This period coincides with the Iron Age Cold Period, spanning 2850–2250 yr BP. Considering *Fagus* ecological characteristics, which favor humid and cool climates, this may be due to its more competitive nature compared to some other plants. Furthermore, its distribution in higher altitudes due to its climate requirements suggests that *Fagus* may have been less affected by human-induced destruction compared to other species [32]. *Quercus* and *Pinus* show relatively low distribution during this period, while *Alnus* is among the lowest. In contrast, the high density of *Artemisia* and Poaceae even in this early period suggests that agricultural and livestock activities were beginning to shape the vegetation composition. Conversely, the relatively high abundance of *Artemisia* and Poaceae even during this early phase suggests that agricultural and pastoral activities had already begun to influence vegetation composition. In Anatolia, fossil pollen studies have shown that between 3200 and 1300 yr BP, the first clear evidence of substantial human impact on vegetation—associated with intensified agricultural practices—was identified from the pollen record of Lake Beyşehir. This interval is therefore referred to as the Beyşehir Occupation Phase (BOP) [33,34,35,36]. During this phase, pollen diagrams typically show marked increases in taxa such as *Fraxinus* sp., *Vitis* sp., *Juglans* sp. and *Olea* sp., providing robust evidence for the influence of human activities on vegetation dynamics [35].

The Roman Climatic Optimum (2200–1500 yr BP) represents one of the warmest phases of the Late Holocene [37,38]. This period is generally characterized by warm and relatively humid climatic conditions across the Mediterranean region [39,40]. An examination of the isopollen maps indicates that at 2000 yr BP, *Fagus* pollen percentages decline both around Lake Sapanca and across the broader study area, whereas *Quercus* becomes more concentrated toward the east, particularly in the vicinity of Lake Iznik. Similarly, *Pinus* pollen percentages increase eastward. *Alnus* remains consistently low during this interval, suggesting that hydrological conditions were not favorable for its development. The continued presence of Poaceae and *Artemisia* throughout the region indicates that anthropogenic influence persisted during this period. Notably, arboreal taxa such as *Pinus*, *Quercus* and *Alnus* reach some of their lowest abundance levels at 2600 and 2000 yr BP. In contrast, herbaceous taxa, particularly Poaceae and *Artemisia*, display relatively high representation during the same intervals. This pattern may reflect a combination of factors: on the one hand, climatic conditions becoming less favorable for the expansion of arboreal taxa, and on the other, increasing anthropogenic pressures such as settlement, agricultural activity, and grazing, leading to the degradation of natural vegetation. The relatively high representation of the Poaceae family around Lake Iznik dating back 2000 years can also be attributed to human activity in the basin. Miebach et al. [18] reported that anthropogenic indicators in the Lake Iznik records became more prominent from the Early Bronze Age onwards, including fruit trees, cultivated plants, and secondary anthropogenic taxa. In this context, it may reflect a landscape shaped by the combined effects of cereal cultivation, tree cultivation, forest clearing, and subsequent vegetation regeneration. The proximity of Lake Iznik to ancient Nicaea, an important urban and economic center during the Roman and Byzantine periods, may further explain the relatively strong human-induced impact observed in this part of the study area. Accordingly, the interval between 2600 and 2000 yr BP can be interpreted as a phase of pronounced environmental transformation in the study area, marked by the combined influence of climatic variability and human activity. The increasing prominence of open land taxa, particularly around Lake Manyas and Lake Iznik, further suggests that this environmental transformation was not spatially uniform, but instead manifested with varying intensity at the basin scale.

The period of 1250 yr BP corresponds to the phase associated with the Migration Period and defined as the Dark Ages Cold Period. Lamb [37] described this period as a cold and unstable climatic phase in Europe; the advance of glaciers in the Alps supports this. The period of 1250 yr BP is the most remarkable in the region in terms of *Pinus* pollen distribution. During this period, *Pinus* rises considerably around Lake Iznik, suggesting that pine forests have become dominant in the inland elevations. *Quercus* also continues to be dominant in the Iznik basin during this period. On the other hand, the momentary increase in *Fagus* pollen data from Lake Sapanca may correspond to a short-term increase in humidity or a local forest recovery during this period. The relatively low levels of *Artemisia* and Poaceae can be attributed to a temporary easing of human pressure or the dominance of climatic conditions that support forest development during this period.

Isopollen maps from 800 yr BP represent a phase in which open-area taxa such as *Artemisia* and Poacea increase again, following the relative increase in forest species observed around 1250 yr BP. This change suggests that, in addition to climatic conditions, agricultural activities, grazing, and settlement pressures may have increased again. However, the fact that tree taxa did not completely decline suggests that this process was not absolute deforestation, but rather a transformation in which forest clearings expanded and human use became more visible. The period of 800 yr BP coincides with the Medieval Warm Period. In Europe, this period, which was effective between 1000 and 700 yr BP, is defined as a phase in which temperatures increased by an average of 0.5–0.8 °C and generally warmer conditions prevailed [37,41]. In the study area, it is observed that arboreal taxa, except for *Alnus*, decrease during this period compared to the previous period, while non-arboreal taxa increase. This change suggests that the warmer conditions of the period supported the spread of herbaceous taxa, while species exhibiting relatively more humid characteristics weakened. However, the high density of *Alnus* during this period suggests that this taxon is related more to local humidity, topographic conditions, and intra-basin hydrological conditions than to regional temperature trends. Furthermore, the fact that hygroscopic taxa are more prominent around Lake Sapanca, while herbaceous taxa are more prominent around Lake Manyas, reveals the influence of topography and regional humidity differences on vegetation distribution during this period. Therefore, 800 yr BP can be considered not only a climatic phase but also a transitional period shaped by the combined effects of local environmental conditions and anthropogenic pressure.

The interval around 400 yr BP corresponds to the Little Ice Age (650–100 yr BP), a period that includes the colder phases associated with Bond Event 0 and is widely recognized as one of the most pronounced cooling intervals of the last two millennia across both the Mediterranean and Europe [42,43,44]. During this period, *Artemisia* reaches its highest abundance, indicating an expansion of open landscapes and a relative decline in forest cover. In addition, the marked decrease in *Fagus* suggests that this taxon—characteristic of cool and humid conditions—became more limited in its representation within the study area compared to earlier periods. This pattern reflects not only climatic deterioration but also the increasing influence of human activities, including deforestation and intensified land use pressure, on vegetation dynamics. Although suitable cool and moist conditions for *Fagus* likely did not disappear entirely, its significant decline indicates that this reduction cannot be attributed solely to climatic variability but must also be considered in the context of anthropogenic disturbance. In contrast, the increase in *Quercus* pollen percentages around Lake Iznik suggests a relative expansion of certain forest elements. Together with the continued presence of *Pinus*, this indicates that arboreal taxa did not completely disappear during this interval. However, the concurrent prominence of open land taxa implies that the landscape did not retain a fully closed forest structure. Instead, it likely evolved into a more heterogeneous and fragmented vegetation mosaic, characterized by the coexistence of arboreal and non-arboreal components.

Assessments based on the generated isopollen maps show that the study area was affected by climate variability during the Late Holocene. This study evaluates the effects of climate change on paleovegetation and examines the role of anthropogenic activities in shaping vegetation dynamics. The IDW interpolation method was applied to visualize the spatial variability of fossil pollen data. While IDW-based interpolation is effective in showing potential spatial continuity between available data points, the reliability of the results largely depends on data density. In this study, the use of only three fossil pollen records from Lake Manyas, Lake Iznik and Lake Sapanca provides an important starting framework for identifying regional patterns. However, this dataset is insufficient to produce a detailed and highly reliable spatial reconstruction of paleovegetation changes in the southeastern Marmara region. Consequently, modelling approaches and spatial analyses based on fossil pollen records require larger and better-distributed datasets to obtain more robust and realistic reconstructions of paleovegetation dynamics.

## 5. Conclusions

The integration of fossil pollen records from lakes Manyas, İznik, and Sapanca provides a regional framework for reconstructing vegetation dynamics in the southeastern Marmara region during the Late Holocene. The resulting isopollen maps reveal a persistent west–east vegetation gradient, reflecting the transitional position of the region between the Mediterranean and Black Sea climatic systems. Open-land taxa such as *Artemisia* and *Poaceae* are more abundant in the western part of the study area, whereas mesophilous and hygrophilous arboreal taxa, particularly *Fagus* and *Alnus*, are more strongly represented in the eastern basins.

The reconstructed vegetation patterns indicate that climatic variability, regional environmental gradients, local ecological conditions, and long-term anthropogenic activities jointly influenced vegetation dynamics over the last 2600 years. During the Iron Age Cold Period (ca. 2600 yr BP), the relatively high abundance of *Fagus* in the Sapanca Basin suggests the persistence of cool and humid conditions, while the occurrence of open-land indicators points to the early influence of human activities. The Roman Climatic Optimum (ca. 2000 yr BP) was characterized by a general decline in arboreal taxa and an increase in herbaceous taxa, reflecting the combined effects of climatic conditions and intensified land use. Around 1250 yr BP, the expansion of *Pinus* and *Quercus*, particularly in the İznik Basin, suggests a phase of enhanced forest development. During the Medieval Warm Period (ca. 800 yr BP), open-land indicators expanded again, consistent with increasing agricultural and pastoral activities, whereas the Little Ice Age (ca. 400 yr BP) was marked by the highest abundance of *Artemisia* and a decline in *Fagus*, indicating the development of a more fragmented landscape under the combined influence of climatic deterioration and intensified human pressure.

The isopollen reconstructions reveal considerable spatial heterogeneity in vegetation change throughout the Late Holocene, with marked differences among individual basins. This finding highlights the importance of adopting spatially integrated approaches when interpreting fossil pollen records from climatically transitional regions. Furthermore, the results demonstrate that vegetation responses were neither synchronous nor uniform across the study area, emphasizing the interacting influences of climate, topography, hydrology, and human land use.

Although IDW interpolation proved useful for visualizing regional vegetation patterns, the reconstructions remain constrained by the limited number of available fossil pollen records. Consequently, the generated maps should be interpreted as generalized representations of regional vegetation trends rather than precise reconstructions of past vegetation cover. The limited spatial coverage partly reflects the underrepresentation of published Turkish fossil pollen datasets in publicly accessible repositories such as the Neotoma Paleoecology Database. Expanding the inclusion of existing fossil pollen records from Türkiye within international open-access databases would substantially improve the spatial resolution and robustness of future paleovegetation reconstructions, while also facilitating broader regional syntheses and advancing open-science initiatives. Future studies incorporating additional well-dated pollen sequences from the Marmara Region will further enhance the reliability of regional paleoecological interpretations.

Overall, the findings demonstrate that vegetation dynamics in the southeastern Marmara region during the Late Holocene were shaped by the complex interaction of climatic fluctuations, regional environmental gradients, local ecological controls, and long-term anthropogenic activities. These results contribute to a better understanding of environmental change in northwestern Anatolia and provide a valuable framework for future regional paleoecological syntheses.

## Figures and Tables

**Table 1:** Study sites used for isopollen maps.

Site No.	Study Area	Latitude/Longitude	Dating Number	Age (ka BP)	References
1	Manyas	40.166461/28.000452	2	4.3–present	[16]
3	Sapanca	40.715736/30.253320	7	2.9–present	[17]
2	Iznik	40.433889/29.533056	9	31–present	[18]

## Data Availability

The fossil pollen datasets used in this study are openly available through the Neotoma Paleoecology Database (https://www.neotomadb.org/). The work of data contributors, data stewards, and the Neotoma community is gratefully acknowledged. This study is based on the first author’s master’s thesis. All processed data and results generated during this study are included within this article.

## Abbreviations

The following abbreviations are used in this manuscript:

GIS	Geographic Information Systems
BOP	Beyşehir Occupation Phase
IDW	Inverse Distance Weighting
